# Integrating spatial transcriptomics and single-cell RNA-sequencing reveals the alterations in epithelial cells during nodular formation in benign prostatic hyperplasia

**DOI:** 10.1186/s12967-024-05212-9

**Published:** 2024-04-23

**Authors:** Xiawei Fei, Jican Liu, Junyan Xu, Hongyan Jing, Zhonglin Cai, Jiasheng Yan, Zhenqi Wu, Huifeng Li, Zhong Wang, Yanting Shen

**Affiliations:** 1 Department of Urology, Qingpu Branch of Zhongshan Hospital Affiliated to Fudan University, Shanghai, 201799 People’s Republic of China; 2 Department of Pathology, Qingpu Branch of Zhongshan Hospital Affiliated to Fudan University, Shanghai, 201799 People’s Republic of China; 3 University of Shanghai for Science and Technology, Shanghai, 200093 People’s Republic of China; 4 Department of Urology and Andrology, Gongli Hospital, the Second Military Medical University, Shanghai, 200135 People’s Republic of China; 5 Department of Urology, Shanghai Ninth People’s Hospital, Shanghai Jiaotong University School of Medicine, Shanghai, 200011 People’s Republic of China

**Keywords:** Benign prostatic hyperplasia, Basal cells, Spatial transcriptomics, Single cell RNA-seq

## Abstract

**Objective:**

Proliferative nodular formation represents a characteristic pathological feature of benign prostatic hyperplasia (BPH) and serves as the primary cause for prostate volume enlargement and consequent lower urinary tract symptoms (LUTS). Its specific mechanism is largely unknown, although several cellular processes have been reported to be involved in BPH initiation and development and highlighted the crucial role of epithelial cells in proliferative nodular formation. However, the technological limitations hinder the in vivo investigation of BPH patients.

**Methods:**

The robust cell type decomposition (RCTD) method was employed to integrate spatial transcriptomics and single cell RNA sequencing profiles, enabling the elucidation of epithelial cell alterations during nodular formation. Immunofluorescent and immunohistochemical staining was performed for verification.

**Results:**

The alterations of epithelial cells during the formation of nodules in BPH was observed, and a distinct subgroup of basal epithelial (BE) cells, referred to as BE5, was identified to play a crucial role in driving this progression through the hypoxia-induced epithelial-mesenchymal transition (EMT) signaling pathway. BE5 served as both the initiating cell during nodular formation and the transitional cell during the transformation from luminal epithelial (LE) to BE cells. A distinguishing characteristic of the BE5 cell subgroup in patients with BPH was its heightened hypoxia and upregulated expression of *FOS*. Histological verification results confirmed a significant association between c-Fos expression and key biological processes such as hypoxia and cell proliferation, as well as the close relationship between hypoxia and EMT in BPH tissues. Furthermore, a strong link between c-Fos expression and the progression of BPH was also been validated. Additionally, notable functional differences were observed in glandular and stromal nodules regarding BE5 cells, with BE5 in glandular nodules exhibiting enhanced capacities for EMT and cell proliferation characterized by club-like cell markers.

**Conclusions:**

This study elucidated the comprehensive landscape of epithelial cells during in vivo nodular formation in patients, thereby offering novel insights into the initiation and progression of BPH.

**Supplementary Information:**

The online version contains supplementary material available at 10.1186/s12967-024-05212-9.

## Introduction

Benign prostatic hyperplasia (BPH) is a highly prevalent condition affecting about 25% of men during their lifetime [1]. It is caused by the progressive growth of the transition zone (TZ) surrounding the proximal urethra, leading to subsequent constriction of the urethral opening [2–4] and resulting in lower urinary tract symptoms (LUTS), particularly common among elderly individuals [1, 2, 5]. Proliferative nodular formation represents a characteristic pathological feature of BPH and serves as the primary cause for prostate volume enlargement and consequent LUTS. Although several cellular processes have been reported to be involved in BPH initiation and development, Its specific mechanism is largely unknown. The contribution of androgen response [6, 7], hypoxia [8, 9], epithelial-mesenchymal transition (EMT) [10–12], etc. in prostatic epithelium to the development of BPH has been acknowledged, highlighting the crucial role of epithelial cells in proliferative nodular formation. However, the current technological limitations hinder the in vivo investigation of BPH patients. The alterations occurring in epithelial cells and their functions during proliferative nodular formation remain unclear.

Spatial transcriptomic (ST) technology, capable of providing a comprehensive spatial gene expression landscape within the architecture of an entire tissue section [13–15], can effectively overcome this limitation. When integrated with single-cell RNA-sequencing (scRNA-seq) technology, it enables the characterization of cellular alterations in tissue structure and facilitates the elucidation of functional linkage between distinct cell clusters and histological structures [15, 16]. Therefore, by integrating these two technologies, we intended to provide a comprehensive understanding of the alterations in epithelial cells and their functions by mimicking the process of proliferative nodular formation in BPH. It will contribute to the identification of novel therapeutic targets.

The prostate epithelium consists of two predominant cell populations: luminal epithelial (LE) cells and basal epithelial (BE) cells. In this study, we observed the transition from LE to BE cell phenotype during proliferative nodular formation in BPH, and identified a distinct subgroup of BE cells, referred to as BE5, which played a crucial role in driving this progression. This subgroup not only serves as the initiating cells during nodular formation but also acts as transitional cells during the transformation from LE to BE cell phenotype. The distinguishing characteristic of the BE5 cell subgroup in patients with BPH was its heightened hypoxia and upregulated expression of *FOS*. Histological verification results confirmed the significant association between c-Fos (coded by *FOS*) expression and key biological processes such as hypoxia and cell proliferation, as well as the close relationship between hypoxia and EMT in BPH tissues. Furthermore, a strong link between c-Fos expression and the progression of BPH was also been validated, underscoring the crucial role played by BE5 cells in the progression of BPH. Additionally, we observed functional differences of BE5 cell subgroup in different nodular phenotypes; primarily functioning in EMT for stromal nodules while playing roles in hypoxia-induced cell proliferation and EMT for glandular nodules. Our findings enhance our understanding of the comprehensive landscape of epithelial cells involved in nodular formation during BPH.

## Methods

### Ethics statement

This study was performed in accordance with the Declaration of Helsinki and was approved by the Ethical Committee of QingPu Branch of Zhongshan Hospital. Written informed consent was obtained from all participants included in this study.

### Patients and sample collection

The prostate tissues were obtained from patients with pathologically confirmed BPH who underwent surgery at the Department of Urology, Qingpu Branch of Zhongshan Hospital Affiliated to Fudan University (Additional file 1: Tables S1 and S2). None of the patients received treatment with 5-alpha reductase inhibitors (5ARIs). Following pathological diagnosis, one tissue sample was subjected to ST sequencing, while the remaining tissue samples were processed for immunofluorescent or immunohistochemical staining. Prostate volume and intravesical prostatic protrusion (IPP) was determined using ultrasonic detection (US).

### Human prostate scRNA-seq data download

Twelves cRNA-seq data matrices of prostate tissues from eight peoples were downloaded from Gene Expression Omnibus (GEO) public database (https://www.ncbi.nlm.nih.gov/geo/) (Additional file 1: Table S3). Five tissues were taken from the benign prostate gland nodules (BPH_GN), four tissues were taken from the benign prostate stroma nodules (BPH_SN), and three tissues were taken from the TZs of the normal prostate tissues (Normal).

### Visium spatial transcriptomics

ST was conducted using the Visium platform (10× Genomics). Cryosection from an OCT-embedded BPH tissue sample was placed on Visium spatial slide. The mRNA molecules bound by printed capture oligos, which contained spatial barcodes on the slide, were converted into cDNA. Subsequently, the cDNA was transferred from the slide for library preparation. Spatial libraries were constructed using the Visium Spatial Library Construction Kit (10× Genomics, PN-1000184), following the manufacturer’s instructions. Finally, sequencing was performed using HiSeq X10 (Illumina) with 150 bp paired-end reads.

### Single-cell RNA-sequencing data analysis

scRNA-seq matrices were loaded in the R package Seurat (Version 3.0.0) for analysis (Additional file 1: Fig. S1A–C). Following data filtering and normalization, integration of the matrices was performed using FindIntegrationAnchors and IntegrateData function (dims = 1:50). Subsequently, standardization of the integrated matrices was carried out to scale scRNA-seq data from 12 matrices, while dimensionality reduction was achieved through RunPCA. Batch effects were removed by employing RunHarmony, and clustering was accomplished via FindNeighbors, FindClusters, and RunUMAP. The FindAllMarkers function was used to analyze the characteristic genes of each cluster (only.pos = TRUE, min.pct = 0.25, logfc.threshold = 0.25). Cell feature gene set scores were calculated using AddModuleScore function [17].

### Spatial transcriptomic analysis

FASTQ files were processed to generate the feature barcode (FB) matrices by using spaceranger software (version 1.1.0) according to Space Ranger pipeline. FB matrices were loaded in the R package Seurat (Version 3.0.0) for subsequent analysis. After data filtering, normalization, and scaling, the FB matrices RunPCA and RunUMAP were used for dimensionality reduction and clustering. The final number of principal components (PCs) was 20, which was determined by the inflection point of the ElbowPlot function. And the *p* value of 20 PCs is less than 0.05 in the JackStrawPlot result. The FindAllMarkers function was used to analyze the characteristic genes of each cluster (only.pos = TRUE, min.pct = 0.25, logfc.threshold = 0.25).

### Cell type decomposition

To obtain the distribution of cell groups in the spatial region, we used the integrated scRNA-seq dataset as reference to perform cell type decomposition in histological structures of the ST slide using the robust cell type decomposition (RCTD) method (doublet_mode = ’full’) [18].

### Single cell gene set enrichment analysis

Single cell gene set enrichment analysis (ssGSEA) on Hallmark gene-set collection (N = 50) were performed using R package irGSEA (Version 1.1.3) (method = c (“singscore”).

### Trajectory analysis

We utilized Monocle to ascertain the lineage differentiation of cell subtypes exhibiting potential developmental relationships. DDRTree was used to learn tree-like trajectories. The branched expression analysis modeling (BEAM) approach was employed to identify differentially expressed genes (DEGs) that determine cellular fate at the specific branch point. A *q* value below 0.05 was considered statistically significant.

### Immunofluorescence (IF) staining

The immunofluorescence (IF) staining protocol employed in this study is consistent with the methodology described by Guo et al. [16]. The primary antibodies used were Anti-c-Fos Rabbit pAb (Servicebio, GB11069) and Anti-Cytokeratin 8 Rabbit pAb (Servicebio, GB11231). Semi-quantitative analysis of fluorescence was performed using ImageJ software. The protocol for analysis was adopted from the previous study [19].

### Immunohistochemical (IHC) staining

The paraffin tissue sections were subjected to dewaxing and rehydration procedures. After performing antigen retrieval, the slides were subjected to blocking using bovine serum albumin (Sango Biotech, Shanghai, China). Subsequently, the slides were incubated overnight at 4 °C with c-Fos Monoclonal antibody (Proteintech, 66590-1-Ig), Vimentin Polyclonal antibody (Proteintech, 10366-1-AP), Anti-E-cadherin antibody (Abcam, ab231303), PCNA Polyclonal antibody (Proteintech, 10205-2-AP), and HIF-1 alpha Polyclonal antibody (Proteintech, 20960-1-AP). Subsequently, the samples were incubated with a secondary antibody of goat anti-rabbit HRP conjugate (Cell Signaling Technology, Beverly, MA, USA) at 25 °C for 1 h. A DAB solution was employed to facilitate brown color development. The regions of interests (ROIs) were analyzed using IHC Profiler [20].

### Statistics

All the analysis was conducted based on R software (version 4.1.3). Student’s t-test, Wilcoxon rank-sum test, and Pearson correlation analysis were utilized in this study. *P* values of less than 0.05 were considered statistically significant (ns, *p* values ≥ 0.05; *, *p* values < 0.05; **, *p* values < 0.01; ***, *p* values < 0.001; ****, *p* values < 0.0001).

## Results

### ST combined with scRNA-seq techniques enabled the identification of spatial characteristics pertaining to distinct cell types within BPH tissue

The scRNA-seq data from twelve samples were utilized to delineate distinct cell populations within prostate tissues (Additional file 1: Table S3). A total of sixteen clusters were identified based on gene expression. The total number of 84,835 cells were annotated as epithelial cells, stromal cells (endothelial cells, fibroblast, and smooth muscle cells [SMC]) and immune cells (T cells, myeloid cells, plasma cells, mast cells, and B cells) based on established marker genes [21]. *PTPRC*− and *EPCAM*+ clusters were identified as epithelial cells, which highly expressed *KRT8* and *KRT18*. *PTPRC*+ clusters were identified as immune cells. Among them, the cluster with high-level expression of *CD3D*, *IL7R*, *CD8A*, and *CD8B* was annotated as T cells; the cluster with high-level expression of *APOE*, *LYZ*, and *IL1B* was annotated as myeloid cells; the cluster with high-level expression of *MZB1* represented plasma cells. The other two remaining *PTPRC*+ clusters were annotated as mast cells expressing *CPA3*, *KIT*, and *TPSAB1*, and a population of B cells expressing *MS4A1*, *CD22*, and *CD79A*. *PTPRC*− and *EPCAM*− clusters were identified as stromal cells, which consisted of endothelial cells (Endo) characterized by *CLDN5*, *SELE*, and *PECAM1* expression, fibroblasts (Fib) expressing *C1S*, *DCN*, and *C7*, and SMC expressing *ACTA2*, *MYH11*, and *RGS5* (Additional file 1: Fig. S1D, E). The UMAP representation of annotated cell types for each group was exhibited in Additional file 1: Fig. S1F. The identification of the top three characteristic genes in each cell type provided further validation for the accurate annotation of these cells (Additional file 1: Fig. S1G).

**Fig. 1:**
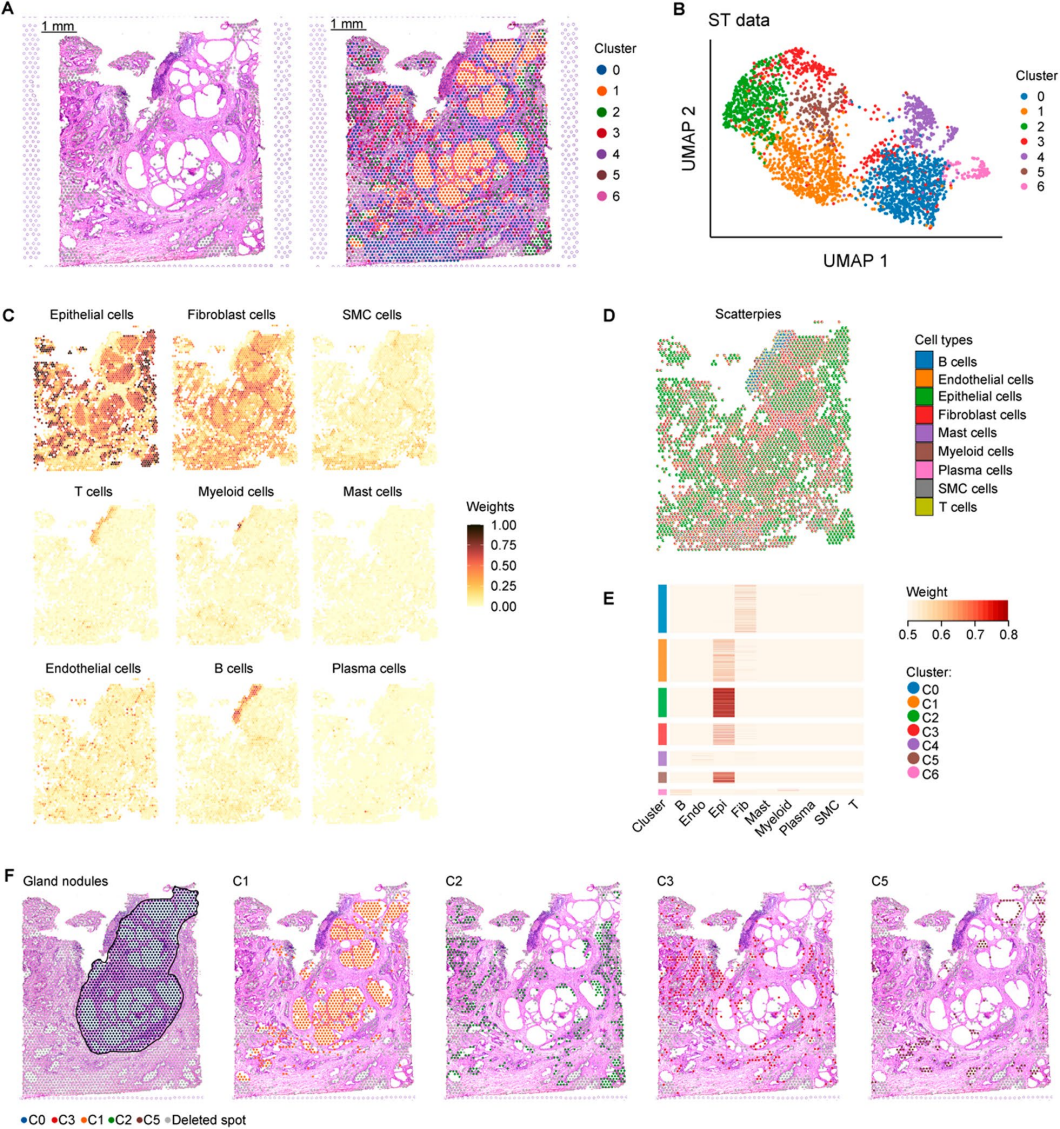
Spatial characteristics pertaining to distinct cell types within BPH tissue**. A** H&E staining of tissue section (left) and mapped with unbiased clustering of ST spots in BPH sample (right). Each cluster is labeled in different color. Scale bar = 1 mm. **B** UMAP plot of 2945 ST spots from a BPH ST data. Each cluster is shown in different color. **(C)** Weight of each cell type in a BPH tissue slide determined using RCTD. **D** Scatterpies of weights for different cell types in a spot in ST data. **E** Heatmap displaying the weight of each cell type for Seurat clusters in ST data. **F** ST spots of nodules, cluster1 (C1), cluster2 (C2), cluster3 (C3), and cluster5 (C5) in the BPH sample, each distinctly labeled in different colors

The spatial distribution of different cell types was assessed by performing ST analysis on a BPH sample consisting of 2945 spots. The ST section of the BPH sample was subjected to histological staining with Hematoxylin and Eosin (H&E) (Fig. 1A). Based on the ST sequencing data, the spatial spots exhibited a classification into seven distinct clusters (C0–C6) (Fig. 1A, B). The RCTD method [18] was employed to integrate the results of ST and scRNA-seq analyses, enabling the identification of predominant cell types within each spatial cluster (Fig. 1C–E). C0 with Fib cells as the predominant component were identified as Fib cluster, distributed both inside and outside the nodule region. C6 were identified as immune cluster due to its high B cell and myeloid cell ratio, predominantly localized outside the nodule region. Notably, four epithelial clusters (C1, C2, C3, and C5) were identified, mainly localized in glandular epithelial regions with epithelial cells as the predominant component. The distribution of C1 spots was predominantly within the nodule region while that of C2, C3, and C5 spots primarily localized outside the nodule region (Fig. 1F), indicating distinct functional roles of these epithelial cells in the formation of nodules.

### ST combined with scRNA-seq techniques enabled the identification of spatial characteristics pertaining to distinct epithelial subtypes within BPH tissue

**Fig. 2:**
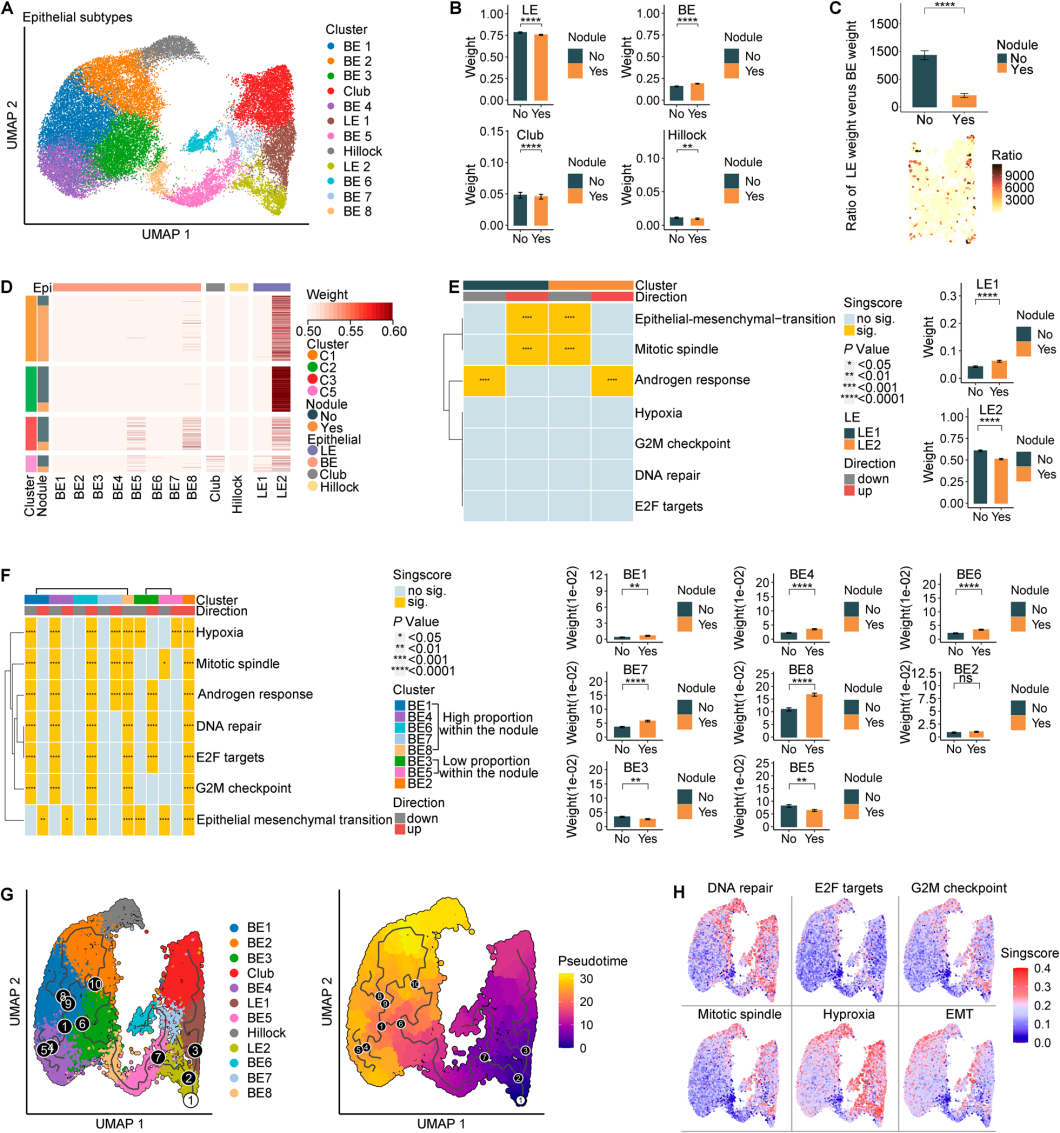
Subsets analysis of epithelial cells. **A** The UMAP coloured according to the epithelial subgroups in scRNA-seq data. **B–F** Subsets analysis of epithelial cells using integrating scRNA-seq and ST data: **B** Bar plot illustrating the weight of LE, BE, Club, and Hillock cell subgroups both within and outside the nodule; **C** Bar plot (up) and BPH tissue slide (down) illustrating the ratio of LE weight versus BE weight; **D** Heatmap displaying the weight of each epithelial cell subgroup for epithelial clusters in ST data; **E** Heatmap displaying the significantly enriched signaling pathways between LE1 and LE2 cells on Hallmark gene-set collection (N = 50) (left), and bar plot illustrating the weight of LE1 and LE2 subgroups both within and outside the nodule (right); **F** Heatmap displaying the significantly enriched signaling pathways among eight BE subgroups on Hallmark gene-set collection (N = 50) (left); Bar plot illustrating the weight of each BE subgroup both within and outside the nodule (right). **G** The UMAP of potential trajectory of all epithelial cells in scRNA-seq data. **H** The UMAP coloured according to the singscore of each Hallmark gene-set in scRNA-seq data. *p* values of the comparison between two variables were determined by a two-sided* t* test. Error bar represented standard error. “**”, *p* value < 0.01; “****”, *p* value < 0.0001

To elucidate the contribution of epithelial cells in the formation of nodules, we first conducted a graph-based sub-clustering analysis using scRNA-seq data to identify distinct epithelial subgroups. Cell-type identification was determined by examining DEGs as well as signature scores using signature gene sets developed from single-cell profiling of normal prostates in a previous study to determine the major epithelial cell subtypes [17] (Additional file 1: Fig. S2A, B). Figure 2A illustrates the identification of eight BE cell subgroups, two LE cell subgroups, one Club cell subgroup, and one Hillock cell subgroup. Clusters exhibiting significantly upregulated BE signature scores and expression of *KRT5*, *KRT14*, and *KRT15* were classified as BE cells. Similarly, clusters displaying significantly elevated LE signature scores along with *KLK2*, *KLK3*, and *ACPP* expression were designated as LE cells. Furthermore, clusters characterized by relatively low LE and BE scores but demonstrating high-expression levels of Club cell marker genes (*SCGB3A1*, *PIGR*, *MMP7*, *CP*, and *LCN2*) and Hillock cell marker genes (*KRT13*, *SERPINB1*, and *CLDN4*), were defined as Club and Hillock cells.

Subsequently, the RCTD method was employed to precisely localize these subgroups within the ST slide (Additional file 1: Fig. S2C). A higher proportion of BE cells but a lower proportion of LE cells were observed within the nodule region compared to outside (Fig. 2B). Additionally, there was a significant decrease in the ratio of LE weight to BE weight within the nodule region compare to that outside the nodule region (Fig. 2C). These findings suggest an increase in BE cell population and depletion of LE cells during nodule formation. Regarding LE cell subgroups, LE1 cell subgroup was predominantly localized in the spatial epithelial region of C5 within BPH tissue, primarily within the nodule region. In contrast, LE2 cell subgroup mainly distributed outside the nodule region in the spatial epithelial regions of C1 and C2 within BPH tissues (Fig. 2D; Additional file 1: Fig. S2D, E). To investigate the functional role of these two LE cell subgroups in silico, we conducted ssGSEA using Hallmark gene set collections (N = 50) on LE1 versus LE2 cell subgroup. Specifically, we focused on Hallmark Androgen Response, Hallmark Epithelial Mesenchymal Transition (EMT), and cell proliferation related Hallmark pathways (including Hallmark DNA repair, Hallmark E2F targets, Hallmark G2M checkpoint, and Hallmark mitotic spindle) that were reported to be closely associated with BPH. We observed a significant upregulated EMT and downregulated Androgen Response signaling in the LE1 cell subgroup, while the opposite pattern was observed for the LE2 cell subgroup (Fig. 2E). Among the BE cell subgroups, namely BE1, BE4, BE6, BE7, and BE8, their distribution in BPH tissues was primarily concentrated within the nodule region. Notably, a significant upregulation of EMT signaling was observed in most of these subgroups (BE1, BE4, and BE6) compared to other BE cell subgroups (Fig. 2F). The enhanced cell proliferation exhibited by the BE6 cell subgroup is particularly noteworthy, as it suggests their potential role as the end contributor cells in nodule formation. In contrast, BE3 and BE5 cell subgroups were predominantly localized outside the nodule region. Notably, both subgroups demonstrated significantly downregulated EMT signaling compared to other BE cell subgroups (Fig. 2F), thereby aligning with the observed alterations in EMT signaling during nodular formation in LE cells. However, it should be noted that there were notable differences in proliferative capacity between these two subgroups. The BE3 cells, predominantly in the spatial epithelial region of C2 and C3 within BPH tissue, exhibited enhanced proliferative capacity, whereas the BE5 cells, mainly in the spatial epithelial region of C3 and C5 within BPH tissue, did not demonstrate a similar effect (Additional file 1: Fig. S2D, E; Fig. 2D, F). Interestingly, we observed a significant augmentation in hypoxia signaling specifically within the BE5 cell population. Hypoxia-induced EMT has been extensively reported in various malignancies, including glioma, prostate cancer (PCa), ovarian carcinoma, lung cancer, and hepatocellular carcinoma [22–25]. Moreover, our research group’s previous investigations have demonstrated the crucial involvement of EMT and the capacity of hypoxia to induce EMT in BPH [8, 26, 27]. In this study, a robust positive correlation was observed between Hypoxia and EMT in epithelial cells (Additional file 1: Fig. S2F). The process of EMT was observed to be associated with the loss of epithelial cell characteristics, such as E-cadherin expression, and an increase in mesenchymal cell features, such as vimentin expression [8, 26, 27]. The immunohistochemical staining results revealed a significant upregulation of vimentin (r = 0.68, *p* value = 0.03) and a notable downregulation of E-cadherin expression (r = -0.84, *p* value = 0.02) in BPH tissues, concomitant with the upregulation of HIF-1a—a hypoxia marker protein (Additional file 1: Fig. S2G). Therefore, we propose that hypoxia acts as the trigger for EMT with the BE5 cell subgroup exhibiting upregulated hypoxia signaling functioning as the initial BE cell population involved in nodular formation.

Finally, in order to further elucidate the evolutionary dynamics of prostate epithelial lineages, we performed pseudo-time cell trajectory analysis on twelve distinct epithelial cell subgroups. Based on the distribution of cell populations within and outside the nodule, we identified the LE2 cell subgroup as the distinct initial point of this evolving trajectory curve. Then, the developmental trajectory illustrating the transition from extranodular to intranodular epithelial cells, as well as from LE to BE cells, was generated (Fig. 2G). The primary objective of our study was to investigate the developmental pathway from the LE2 subgroup, predominantly located outside the nodule region, to the nodular formation end effector BE6 subgroup, primarily situated within the nodule region (Fig. 2G; Additional file 1: Fig. S2H), where enhanced hypoxia, EMT, and cell proliferation signals were observed (Fig. 2H). We observed that BE5 cells with upregulated hypoxia signaling, characterized by high expression levels of *FOS* and *JUN* (Additional file 1: Fig. S2I), not only functioned as initiating BE cells in nodular formation processes but also acted as transitional intermediates during the transformation from LE to BE cells. The *FOS* and *JUN* genes encode the FOS and JUN protein families, which form a heterodimeric complex known as activator protein-1 (AP-1), functioning as a transcription factor. The FOS-target factor gene set and JUN-target factor gene set obtained from the Transcriptional Regulatory Relationships Unraveled by Sentence-based Text mining v2 (TRRUST v2) database [28] were subjected to gene set enrichment analysis (GSEA). The results demonstrated a significant upregulation of both target factor gene sets in BE5 cells compared to other BE cells (Additional file 1: Fig. S2J, K), indicating the heightened AP-1 transcriptional activity exhibited by BE5 cells.

### Pseudo-time cell trajectories depicting the transition from LE to BE cells, as well as from extranodular BE cells to intranodular BE cells

**Fig. 3:**
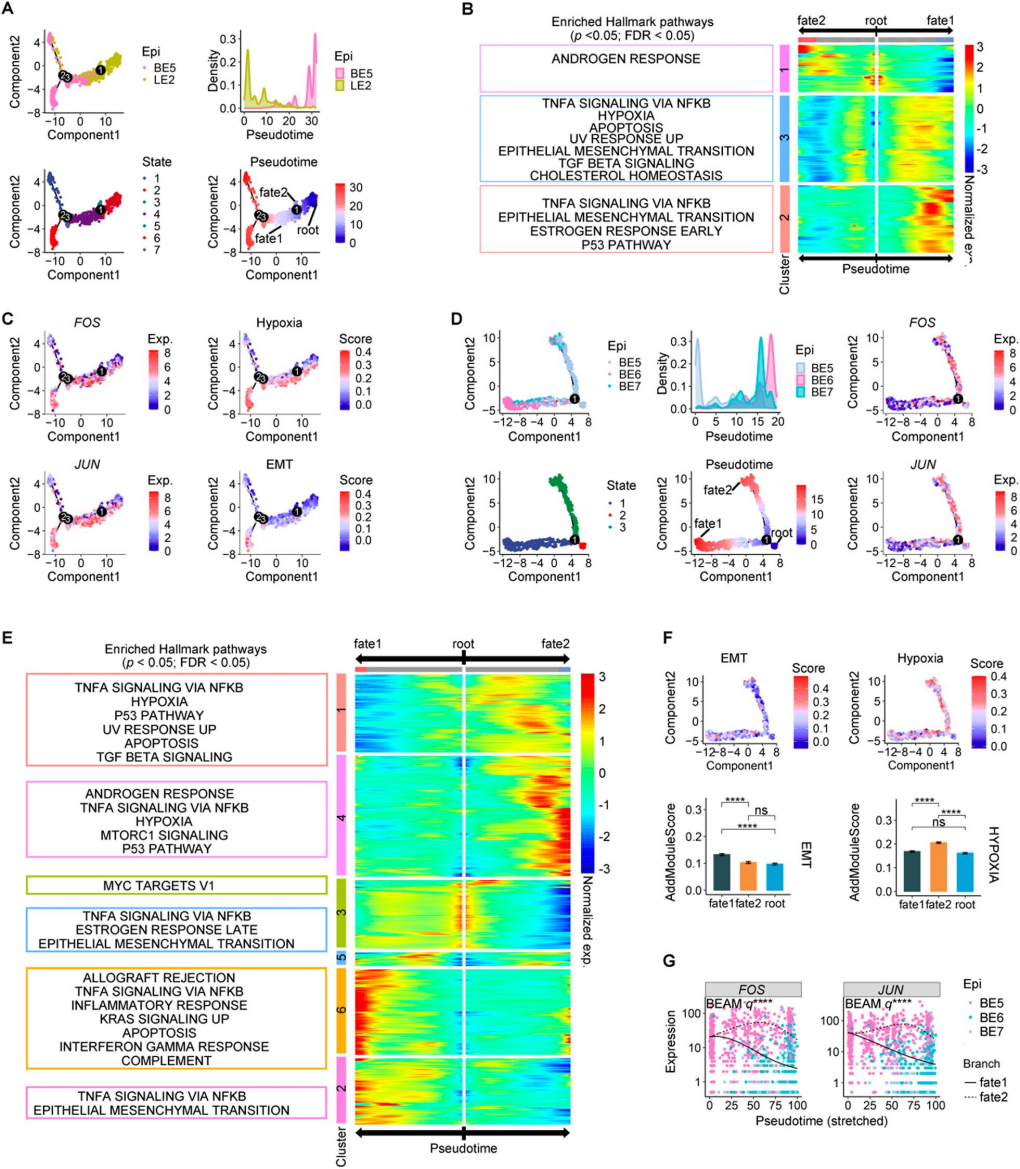
Pseudo-time cell trajectories depicting the alterations in epithelial cells during nodular formation using scRNA-seq. **A** Pseudo-time cell trajectory of LE2 and BE5 cells colored by epithelial cell subtypes (Epi), state, and pseudo-time. **B** Heatmap showing the selected DEGs (*q* value < 1e-04) which was clustered into three profiles as well as the significant enriched Hallmark pathways for each cluster (*p* value < 0.05; FDR < 0.05). **C** Pseudo-time cell trajectory of LE2 and BE5 cells colored by the expression levels of *FOS* and *JUN* and the scores of Hypoxia and EMT signaling pathways. **D **Pseudo-time cell trajectory of BE5, BE6, and BE7 cells colored by epithelial cell subgroups (Epi), state, pseudo-time, and the expression levels of *FOS* and *JUN*. **E** Heatmap showing the selected DEGs (*q* value < 1e-04) which was clustered into six profiles as well as the significant enriched Hallmark pathways for each cluster (*p* value < 0.05; FDR < 0.05). **F** Pseudo-time cell trajectory of BE5, BE6, and BE7 cells colored by the scores of Hypoxia and EMT signaling pathways (up); Bar plot illustrating the scores of Hypoxia and EMT signaling pathways among root, fate 1, and fate 2 cells (down). **G** Dot plots of dynamic expression of *FOS* and *JUN* along two cell fates. Hypoxia and EMT gene sets were scored using AddModuleScore method. The *p* values of the comparison between two variables were determined using a two-sided Wilcoxon test. Error bar represented standard error. “****”, *p* value < 0.0001

While the transition from a LE to BE cells has been demonstrated in certain epithelial lesions, such as breast cancer [29–35], there is limited evidence supporting this phenomenon in BPH. To elucidate the evolutionary dynamics underlying the transition from LE to BE cells in BPH, we constructed a pseudo-time cell trajectory for the LE2 and BE5 cell subgroups (Fig. 3A). The evolutionary trees depicted the trajectory from LE2 to BE5, simulating the transformation process from LE to BE cells. LE2 cells underwent bifurcation into either fate 1 or fate 2 branches, wherein fate 1 led to the transition of LE2 cells into BE5 cells while fate 2 comprised of persisting LE2 cells. Therefore, we proposed that branch point 1 played a pivotal role in determining the differentiation of LE cells into BE cells. We identified DEGs in this branch point, which played a crucial role in determining the transition from the root to either fate 1 or fate 2. DEGs with *q* values less than 1e-04 were selected to construct a heatmap and classified into three clusters (Fig. 3B). Enrichment analysis based on Hallmark gene sets (N = 50) was conducted for DEGs of each cluster, revealing that DEGs in cluster 1, upregulated in fate 2, exhibited significant enrichment in Androgen response signaling pathways. Conversely, DEGs in clusters 2 and 3, upregulated in fate 1, demonstrated notable enrichment in Hypoxia and EMT signaling pathways. Consistent with Liu’s findings in breast cancer [29], a significant enhancement of the EMT signal was also observed during the transition from LE to BE phenotype in BPH. Moreover, our results showed that the signaling of Hypoxia was persisting along the trajectory of fate 1, while the signaling of EMT was evident among clustered cells at the end of the evolutionary trees, indicating the role played by Hypoxia in inducing EMT (Fig. 3C). These findings suggest the crucial roles of Hypoxia induced EMT in the transition from LE to BE cells.

To elucidate the evolutionary dynamics of the alterations in BE cells during nodular formation, we constructed pseudo-time cell trajectory for the BE5, BE6 and BE7 cell subgroups. The evolutionary trees depicted the transformation process from extranodular initial cells (BE5 cells) to intranodular main end contributor cells, which were responsible for nodule formation (BE6 cells) (Fig. 3D). The fate of BE5 cells diverged into two paths: along fate 1 trajectory, some transformed into BE6 cells, while others persisted as high *FOS* and *JUN* expressing BE5 cells along fate 2 trajectory. A heatmap was generated using DEGs of branch point 1 with *q* values less than 1e-04 (Fig. 3E). Enrichment analysis based on Hallmark gene sets (N = 50) was revealed that DEGs in clusters 2 and 6, upregulated in fate 1, exhibited significant enrichment in EMT signaling pathway. DEGs in clusters 1 and 4, upregulated in fate 2, demonstrated notable enrichment in Hypoxia signaling pathway. We further compared Hypoxia and EMT signaling between cells in fate1 and root (Fig. 3F). The results revealed a significantly higher activation of the EMT signaling pathway in fate 1 cells compared to root cells; however, no significant difference was observed in the levels of Hypoxia signaling between fate 1 and root cells. Furthermore, we compared the Hypoxia and EMT signaling pathways between fate 2 cells and root (Fig. 3F). The findings demonstrated a significantly higher activation of Hypoxia signaling pathways in fate 2 cells when compared to root cells. However, when comparing with fate 1 cells, we observed a notably higher activation of the Hypoxia pathway but a lower activation of the EMT pathway in fate 2 cells. Additionally, elevated expression levels of *FOS* and *JUN* were detected in fate 2 cells when compared to those in fate 1 cells (Fig. 3G). Previous studies have demonstrated that under hypoxic conditions, the AP-1 complex can be activated, thereby promoting cell proliferation through the activation of JNK or ERK signaling pathways [36]. To summarize, we propose that during the process of nodule formation, a specific subset of BE5 cells undergoes EMT and transforms into the end contributor BE6 cells, while another distinct subset characterized by persistent Hypoxia signals and high *FOS* and *JUN* expression possibly serves as a reservoir for the self-renewal of BE5 cells.

### BE5 cell subgroup enhancing the progression of BPH

**Fig. 4:**
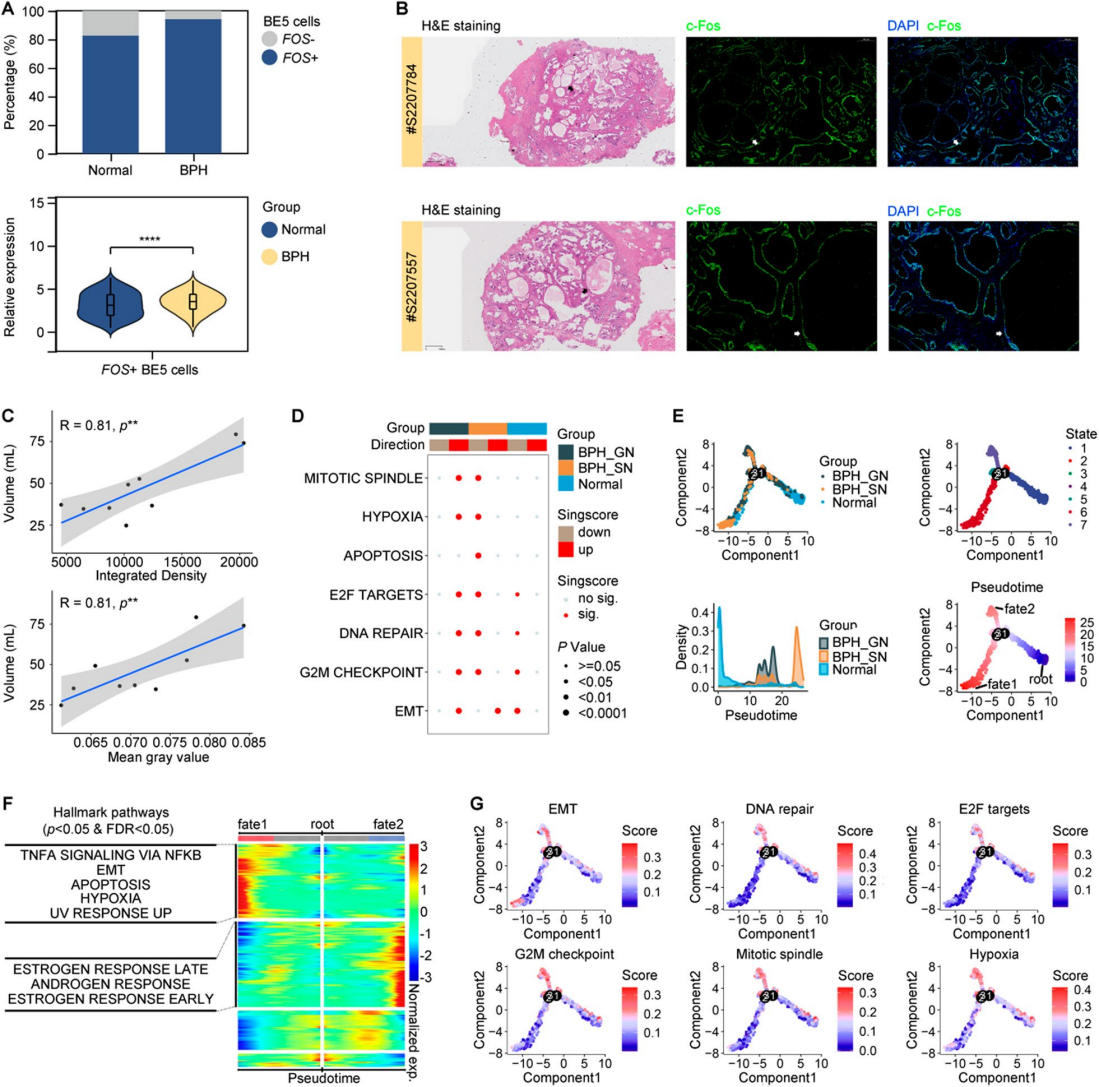
BE5 cell subgroup enhancing the progression of BPH**. A** Bar plot illustrating the percentage of *FOS*− and *FOS*+ BE5 cells in the total of normal and BPH BE5 cells (up); Violin plots depicting the expression levels of *FOS* in normal and BPH *FOS*+ BE5 cells (down). **B** Analysis of c-Fos expression by anti-c-Fos IF (green) in prostate tissues of BPH patients (nuclei counterstained with DAPI). The scale bar was set at 500 μm for H&E images and 100 μm for IF images. **C** Scatter plot illustrating the Pearson correlation between prostate volume and both the integrated density and mean gray value of c-Fos. **D** Heatmap displaying the significantly enriched signaling pathways among normal, BPH_GN, and BPH_SN BE5 cells on Hallmark gene-set collection (N = 50) in scRNA-seq data. **E** Potential trajectory of normal, BPH_GN, and BPH_SN BE5 cells colored by group, state, and pseudo-time in scRNA-seq data. **F** Heatmap showing the selected DEGs (*q* value < 1e-04) which was clustered into four profiles as well as the significant enriched Hallmark pathways for the clusters in scRNA-seq data (*p* value < 0.05; FDR < 0.05). **G** Potential trajectory of normal, BPH_GN, and BPH_SN BE5 cells colored by the scores of Hypoxia, EMT, and cell proliferation related Hallmark pathways in scRNA-seq data

To elucidate the role of the BE5 cell subgroup in the progression of BPH, we initially compared the expression levels of its characteristic genes, *FOS* and *JUN*, in BE5 cells derived from BPH and normal tissue samples. We observed that BE5 cells derived from BPH tissue samples exhibited higher *FOS* expression but relatively lower *JUN* expression compared to those derived from normal tissue samples (Additional file 1: Fig. S3A). Although the proportion of BE5 cells in epithelial cells in BPH tissues did not show a significant difference compared to that in normal tissues (Wilcoxon test, *p* value > 0.05; Additional file 1: Fig. S3B, C), a higher number of *FOS*+ BE5 cells were observed in BPH tissues than in normal prostate tissues (chi-square test, *p* value < 0.0001; Fig. 4A). Furthermore, the expression level of *FOS* was significantly elevated in these BPH *FOS*+ BE5 cells when compared to normal *FOS*+ BE5 cells (Wilcoxon test,* p* value < 0.0001; Fig. 4A). The GSEA results revealed that BE5 cells in BPH tissues exhibited enhanced FOS transcriptional activities, while their JUN transcriptional activities remained unaltered compared to other BE cells (Additional file 1: Fig. S3D, E). These results highlight a close relationship between *FOS* expression in BE5 cells and BPH. To further investigate this association, clinical histological experiments were performed. The results revealed that c-Fos protein, encoded by *FOS*, exhibited expression in both LE cells and BE cells of prostate tissue; however, it predominantly manifested in BE cells of BPH tissues (Additional file 1: Fig. S4A, B). The expression level of c-Fos was found to be significantly positively correlated with the expression level of HIF-1α, a hypoxia marker protein (r = 0.80, *p* = 5.1e-3; Additional file 1: Fig. S4C). Additionally, the PCNA expression, a cell proliferation marker protein, was significantly upregulated in the epithelium exhibiting high c-Fos expression compared to that with relatively low c-Fos expression in BPH tissues (*t*-test: p = 0.03; Additional file 1: Fig. S4D). These findings further support the presence of a BE5 cell subgroup characterized by elevated FOS expression and hypoxia in BPH tissues. Additionally, a significant positive correlation between c-Fos expression and cellular proliferation is proposed, emphasizing the potential capacity of the BE5 cell subgroup in self-renewal. Furthermore, through the integration of medical imaging examination data obtained from patients with BPH, we have elucidated the correlation between c-Fos expression and both the severity and progression of clinical symptoms in BPH, utilizing indicators such as prostate volume and IPP. Our findings revealed a significant positive association between c-Fos expression levels and prostate volume (r = 0.81, *p* = 0.003; Fig. 4B, C), while also demonstrating that patients with a high degree of IPP (> 10 mm) exhibited higher c-Fos expression compared to those with a low degree of IPP (≤ 10 mm) (t-test: *p* = 2.7e−3; Additional file 1: Fig. S5). Notably, IPP exhibited a significant correlation with increased prostate volume, heightened obstructive symptoms, and reduced peak urinary flow rate, thereby presenting objective clinical utility in symptom evaluation and progression prediction [37]. These findings collectively underscore the crucial role played by BE5 cells in BPH.

Additionally, according to the different constituent cell types, proliferative nodules can be divided into stromal nodules and glandular nodules. Stromal nodules mainly consist of stromal, while glandular nodules mainly consist of glandular epithelium [4]. However, it is noteworthy that both phenotypes of these two proliferative nodules frequently coexist within the same hypertrophied prostate [5], implying a potential interrelationship between the processes underlying their formation. In this study, we aimed to elucidate this phenomenon from the perspective of BE5 cells. We initially performed ssGSEA using Hallmark gene set collections (N = 50) on the BE5 cell subgroups of normal, glandular nodules (BPH_GN) and stromal nodules (BPH_SN) tissues to investigate their functions in silico. We observed a significant upregulation EMT signaling in both the BE5 cell subgroups of BPH_GN and BPH_SN, as well as a significant upregulation of Hypoxia and cell proliferation related signaling pathways in BE5 cell subgroups of BPH_GN (Fig. 4D). The findings suggest that within stromal nodules, BE5 subgroup primarily functions in EMT, while it plays roles in both cell proliferation and EMT within glandular nodules due to its upregulated Hypoxia signaling. Additionally, we constructed a pseudo-time cell trajectory for the BE5 cell subgroups of normal, BPH_GN, and BPH_SN to simulate the alterations of BE5 cells in the formation process of stromal and glandular nodules. The evolutionary trees depicted the successive alternation in the functions of BE5 cells in the progression of both two types of BPH (Fig. 4E). Normal BE5 cells underwent bifurcation at branch point 3, giving rise to fate 1 or fate 2 branches. Fate 1 resulted in the transition of normal BE5 cells into BPH_SN BE5 cells, while fate 2 led to the transition of normal BE5 cells into BPH_GN BE5 cells. We identified DEGs at the branch point 3, which exhibited distinct patterns of transformation from the root to either fate 1 or fate 2 (Fig. 4F). Enrichment analysis based on Hallmark gene sets (N = 50) was performed, revealing that upregulated DEGs in fate 2 exhibited significant enrichment in Androgen response and Estrogen response early and late signaling pathways. Conversely, upregulated DEGs in fate 1 demonstrated notable enrichment in Hypoxia and EMT signaling pathways. The signaling of Hypoxia and cell proliferation-related pathways were enhanced at the end of fate 2, while the signaling of EMT was enhanced at both ends of the two cell fates (Fig. 4G). The present findings provide further evidence supporting the primary involvement of BE5 in EMT during stromal nodular formation, while also highlighting its roles in hypoxia-induced cell proliferation and EMT during glandular nodular formation. Additionally, the top three DEGs at branch point 3 were *SCGB1A1*, *LTF*, and *MMP7*. These DEGs exhibited upregulation in fate 2 but downregulation in fate 1, suggesting that BE5 cells in glandular nodules displayed distinct club-like characteristics (Additional file 1: Fig. S6).

## Discussions

BPH presents limited drug targets, with alpha-blockers and 5-ARIs currently serving as the primary clinical treatments. Among them, only 5-ARIs have been demonstrated to effectively reduce prostate volume and delay the progression of BPH [38, 39]. However, it can lead to adverse effects such as sexual dysfunction including erectile difficulties and abnormal ejaculation [40]. Additionally, in 25%-30% of patients, there is no observed improvement in prostate volume or LUTS following treatment with 5-ARIs, while approximately 5%-7% of patients experience a progressive worsening of symptoms necessitating surgical intervention [38, 40]. Therefore, the identification of novel drug targets for BPH is of paramount importance, thereby augmenting the therapeutic repertoire to benefit a larger cohort of patients. The proliferative nodular formation represents the classical pathological hallmark of BPH, serving as the primary etiology for prostate volume enlargement and subsequent LUTS. A comprehensive understanding of the intricate processes underlying proliferative nodules holds immense clinical significance in terms of identifying novel therapeutic targets for BPH. Previous studies have indicated that, apart from LE cells which are targeted by 5-ARIs, other cell types within the prostate have been implicated in this pathogenesis [10, 12, 41–43], highlighting their potential as therapeutic targets for BPH. However, due to technological limitations, their contributions have yet to be validated through in vivo investigations conducted on patients with BPH.

ST technology can effectively overcome this limitation [15, 16]. The technique facilitates the assessment of cellular changes both within and outside the nodule structure in prostate tissue, thereby contributing to a comprehensive understanding of the functional interplay between cells and nodular formation. When integrated with scRNA-seq technology, its resolution can be enhanced to the single-cell level. By leveraging these two complementary methodologies, we identified a fibroblast cluster and four distinct epithelial clusters. The spatial distribution of the fibroblast cluster encompassed both intra- and extra-nodular regions, suggesting little difference between fibroblasts residing within and outside the nodule. However, noticeable differences were observed between intra- and extra-nodular epithelial cells. Among the four epithelial clusters, one cluster predominantly localized within the nodule while the remaining three clusters primarily distributed in extra-nodular regions. These findings underscored the pivotal involvement of epithelial cells in nodular formation.

Therefore, we employed a pseudo-time trajectory to simulate the formation of nodules in patients with BPH, enabling a comprehensive analysis of the characteristic and functional changes occurring in epithelial cells throughout this process. After sub-clustering, the epithelial cells were clustered into twelve subgroups. The precise identification of the epithelial subgroup that served as the initiation or termination points for the pseudo-time trajectory was of utmost significance, primarily guided by the following factors. Firstly, we considered the distribution of epithelial subgroups both within and outside the nodule region. The predominantly nodule-region-distributed epithelial subgroup was selected as the termination point of the pseudo-time trajectory, while the predominantly non-nodule-region-distributed group was chosen as its initiation point. Secondly, we took into account cell functionality. Although various cellular pathways have been reported to be involved in BPH, such as nuclear factor-kappa B (NF-kappaB) signaling pathway, TGF-β1 signaling pathway, and inflammation related pathways, EMT is commonly considered at the downstream of these pathways in BPH [44–46]. BPH has been recognized as a disease characterized by the accumulation of mesenchymal-like cells derived from the prostatic epithelium [47]. Therefore, we selected the subgroups with a high EMT signal as the pseudo-time trajectory endpoint. BPH is an age-related disease and hypoxia often occurs in elderly individuals. Additionally, Hypoxia-induced EMT has been extensively reported [8, 22–27]. Thus, we designated the cell population exhibiting upregulated hypoxia signaling as the initial point of the pseudo-temporal trajectory. Lastly, we took into consideration the number of cells within and outside nodules. We observed a higher abundance of BE cells within nodules compared to those outside, while the presence of LE cells within nodules was lower than that outside. This finding suggests the potential proliferation of BE cells, which aligns with the pathological characteristics associated with the increasing numbers of epithelial cells in nodule formation. Consequently, our primary focus lied in elucidating the pseudo-time trajectory with the highly proliferative BE cells as the terminal point.

In the established pseudo-time trajectory in this study, the LE2 cell subgroup predominantly localized outside the nodule at one end of the cell trajectory, while the BE6 cell subgroup primarily distributed within the nodule and exhibited significant activation of EMT and cellular proliferation signaling at the other end. Therefore, we selected the LE2 cell subgroup as the initiation point for the established pseudo-time trajectory and focused on investigating the branch ending with the BE6 cell subgroup. Within this branch, we observed two distinct processes, with the first involving a transformation from LE to BE cells. While the transition from a LE to BE cells has been demonstrated in certain epithelial lesions [29–35], there is limited evidence supporting this phenomenon in BPH. In recent years, the increased utilization of single-cell transcriptomics has unveiled a distinct subset of LE cells that co-express both LE and stem cell-associated genes in the mouse prostate [48–52]. However, the mechanisms underlying their amplification in BPH remain poorly understood and the available evidence to confirm the possibility of direct transformation from LE to BE cells is also insufficient [53]. In this study, we employed pseudo-time trajectory analysis as an innovative approach to observe the dynamic transition of LE cells into BE cells during nodular formation in patients with BPH. Consistent with Liu’s findings in breast cancer [29], a significant enhancement of the EMT signal was also observed during the transition from LE to BE phenotype in BPH. To our knowledge, this represents the first documented observation of LE-to-BE cell transformation during nodular formation in BPH patients. Furthermore, we observed a distinct BE5 cell subgroup exhibiting high hypoxia signaling, which was identified as transition cells during this process. The second process within the LE2-to-BE6 branch involved a transition from extranodular to intranodular BE cells (BE5-to-BE6). During this process, hypoxia-induced EMT was notably noticed, and the BE5 cell subgroup, exhibiting high hypoxia signaling, was identified as the initial cells that facilitated the transformation of extranodular BE cells into intranodular end-contributor BE cells for nodular formation. The BE5 cell subgroup was characterized by the high expression of *FOS* and *JUN*, which encode proteins that form the heterodimeric AP-1 transcription factor. Furthermore, we observed a significantly elevated level of *FOS* expression and enhanced FOS transcriptional activities in the BPH BE5 cell subgroup compared to normal BPH BE5 cell subgroups. Previous studies have demonstrated that under hypoxic conditions, the AP-1 complex can be upregulated and activated, thereby affecting cell proliferation and EMT [36, 54]. Therefore, we postulate that BE5 cells with high *FOS* expression may play a pivotal role in the progression of BPH. Prostate volume and IPP were utilized as objective indicators to assess BPH progression and symptoms. Our findings revealed that BPH patients exhibiting elevated c-Fos expression displayed larger prostate volume and more severe IPP, thus highlighting the significant contribution of the BE5 cell subgroup to BPH. Additionally, these findings have focused on the involvement of BE cells rather than LE cells in BPH, thereby enhancing our comprehension of the heterogeneous histological response of prostate glands to androgen deprivation, such as 5ARI treatment. This discovery offers a potential novel therapeutic target for BPH that operates independently of androgen receptor signaling.

Furthermore, in this study, we aimed to clarify the correlation between glandular and stromal nodular formation by utilizing pseudo-time trajectory analysis on BE5 cells. We noticed that upregulated EMT signaling occurred in the BE5 cells in both types of nodules, while high cell proliferation signaling was only observed in the BE5 cells in glandular nodules. Notably, BE5 cells in glandular nodules were identified to be displayed distinct characteristics of club cells. Considering their high cell proliferation capacity resembling that of club cells in prostate tissues [53, 55, 56], we regarded them as club-like BE cells.

This study still has several limitations. Firstly, the ST data can only be accessed within the cells of each spot and are lost at intervals between every two spots [16]. With advancements in ST technology, it is anticipated that higher resolution and shorter interval distances may be achieved in future studies. Secondly, the limited sample size used for ST analysis in this study restricts the generalizability of our conclusions. Nevertheless, to mitigate the impact caused by this small sample size in ST sequencing, we integrated ST with scRNA-seq data from five BPH samples and three normal prostate samples to enhance the reliability of our findings. However, future research should prioritize conducting multi-center clinical trials with larger sample sizes to establish individual patient trajectories [57] and thereby validate the significance of *FOS* in the development of BPH nodules. This will lay a robust groundwork for further applications in constructing BPH animal models and conducting treatment research.

Our findings have unveiled the comprehensive landscape of epithelial cells during nodular formation in vivo in patient with BPH, thereby offering novel insights into the initiation and progression of BPH, holding potential therapeutic implications targeting.

### Supplementary Information

**Additional file1. Table S1. **General information of samples used for Immunofluorescent staining. **Table S2. **General information of samples used for Immunohistochemical staining. **Table S3. **General information of samples used for ST and single cell RNA sequencing. **Figure S1. **The atlas of single-cell and spatial transcriptomics in prostate tissues (n=13). **Figure S2.** Subsets analysis of epithelial cells. **Figure S3.** Characteristic of BE5 subgroup in BPH tissue. **Figure S4.** Localization and expression of *FOS* within BPH tissues. **Figure S5.** Bar plot illustrating the percentage of positive (100-negative) c-Fos expression in both IPP>10mm and IPP≤10mm BPH patients. **Figure S6.** Dot plots of dynamic expression of top four DEGs along two cell fates at the branch point 3 in the pseudo-time cell trajectory for the normal, BPH_GN, and BPH_SN BE5 cells.

## Data Availability

ST data were deposited at Gene Expression Omnibus GSE242249. The datasets supporting the conclusions of this article are included within the article and its additional files. Further inquiries can be directed to the corresponding authors.
