# Supplementary material for: Integrating spatial transcriptomics and single-cell RNA-sequencing reveals the alterations in epithelial cells during nodular formation in benign prostatic hyperplasia
Source: J Transl Med. 2024 Apr 23;22:380. doi: 10.1186/s12967-024-05212-9 (PMC11036735; doi:10.1186/s12967-024-05212-9)
Supplement: Supplementary file 1 — Additional file1. Table S1. General information of samples used for Immunofluorescent staining. Table S2. General information of samples used for Immunohistochemical staining. Table S3. General information of samples used for ST and single cell RNA sequencing. Figure S1. The atlas of single-cell and spatial transcriptomics in prostate tissues (n=13). Figure S2. Subsets analysis of epithelial cells. Figure S3. Characteristic of BE5 subgroup in BPH tissue. Figure S4. Localization and expression of FOS within BPH tissues. Figure S5. Bar plot illustrating the percentage of positive (100-negative) c-Fos expression in both IPP>10mm and IPP≤10mm BPH patients. Figure S6. Dot plots of dynamic expression of top four DEGs along two cell fates at the branch point 3 in the pseudo-time cell trajectory for the normal, BPH_GN, and BPH_SN BE5 cells. [file 12967_2024_5212_MOESM1_ESM.docx]

**Supplementary materials**

**Tables:**

**Supplementary Table S1.** General information of samples used for Immunofluorescent staining

| **Patients** | **Volume(mL)** | **5ARIs**  **treatment** | **Integrated density** | **Area** | **Mean gray value^*1^** |
| --- | --- | --- | --- | --- | --- |
| S2206078 | 52.624 | No | 11326 | 146906 | 0.077096919 |
| S2206480 | 24.71 | No | 10182 | 166244 | 0.061247323 |
| S2207539 | 34.68 | No | 6480 | 88545 | 0.073183127 |
| S2207556 | 49.19 | No | 10351 | 157924 | 0.065544186 |
| S2207557 | 74.07 | No | 20351 | 241519 | 0.084262522 |
| S2207896 | 35.217 | No | 8717 | 138787 | 0.062808476 |
| S2207773 | 36.65 | No | 12414 | 180904 | 0.068622032 |
| S2207784 | 79.29 | No | 19681 | 251428 | 0.078276882 |
| S2208372 | 37.15 | No | 4553 | 64558 | 0.070525729 |

**^*1^** Mean gray value=Integrated density/Area

**Supplementary Table S2.** General information of samples used for Immunohistochemical staining

| **ID** | **5ARIs treatment** | **Volume (mL)** | **IPP (mm)** |
| --- | --- | --- | --- |
| #229_1 | No | 62.30016 | >10 |
| #229_2 | No | 75.66260832 | >10 |
| #229_3 | No | 65.58376448 | >10 |
| #229_4 | No | 87.03968 | >10 |
| #229_5 | No | 80.496 | >10 |
| #229_6 | No | 30.43579968 | <10 |
| #229_7 | No | 25.24176096 | <10 |
| #229_8 | No | 92.21121728 | <10 |
| #229_9 | No | 54.25056 | <10 |
| #229_10 | No | 44.7174 | <10 |

**Supplementary Table S3.** General information of samples used for ST and single cell RNA sequencing

| **Patients** | **Sample** | **Matrix ID** | **Group** | **Nodule** | **Detection** | **5ARIs**  **treatment** | **Cells (spots) after filtering** |
| --- | --- | --- | --- | --- | --- | --- | --- |
| P1 | BPH283_GN | GSM5252126 | BPH | Yes(GN) | scRNA-seq | No | 4,483 |
|  | BPH283_SN | GSM5252127 | BPH | Yes(SN) | scRNA-seq | No | 2,964 |
| P2 | BPH327_GN | GSM5252128 | BPH | Yes(GN) | scRNA-seq | No | 5,389 |
|  | BPH327_SN | GSM5252129 | BPH | Yes(SN) | scRNA-seq | No | 8,744 |
| P3 | BPH340_GN | GSM5252130 | BPH | Yes(GN) | scRNA-seq | No | 7,364 |
|  | BPH340_SN | GSM5252131 | BPH | Yes(SN) | scRNA-seq | No | 7,523 |
| P4 | BPH389_GN | GSM5252132 | BPH | Yes(GN) | scRNA-seq | No | 6,000 |
|  | BPH389_SN | GSM5252133 | BPH | Yes(SN) | scRNA-seq | No | 13,596 |
| P5 | BPH511_GN | GSM5252134 | BPH | Yes(GN) | scRNA-seq | No | 9,993 |
| D17 | D17 | GSM5252458 | Normal | - | scRNA-seq | - | 6,125 |
| D27 | D27 | GSM5252460 | Normal | - | scRNA-seq | - | 7,612 |
| D35 | D35 | GSM5252462 | Normal | - | scRNA-seq | - | 5,688 |
| A4 | A4 | GSE242249 | BPH | Yes | ST | No | 2,945 |

**Figures**


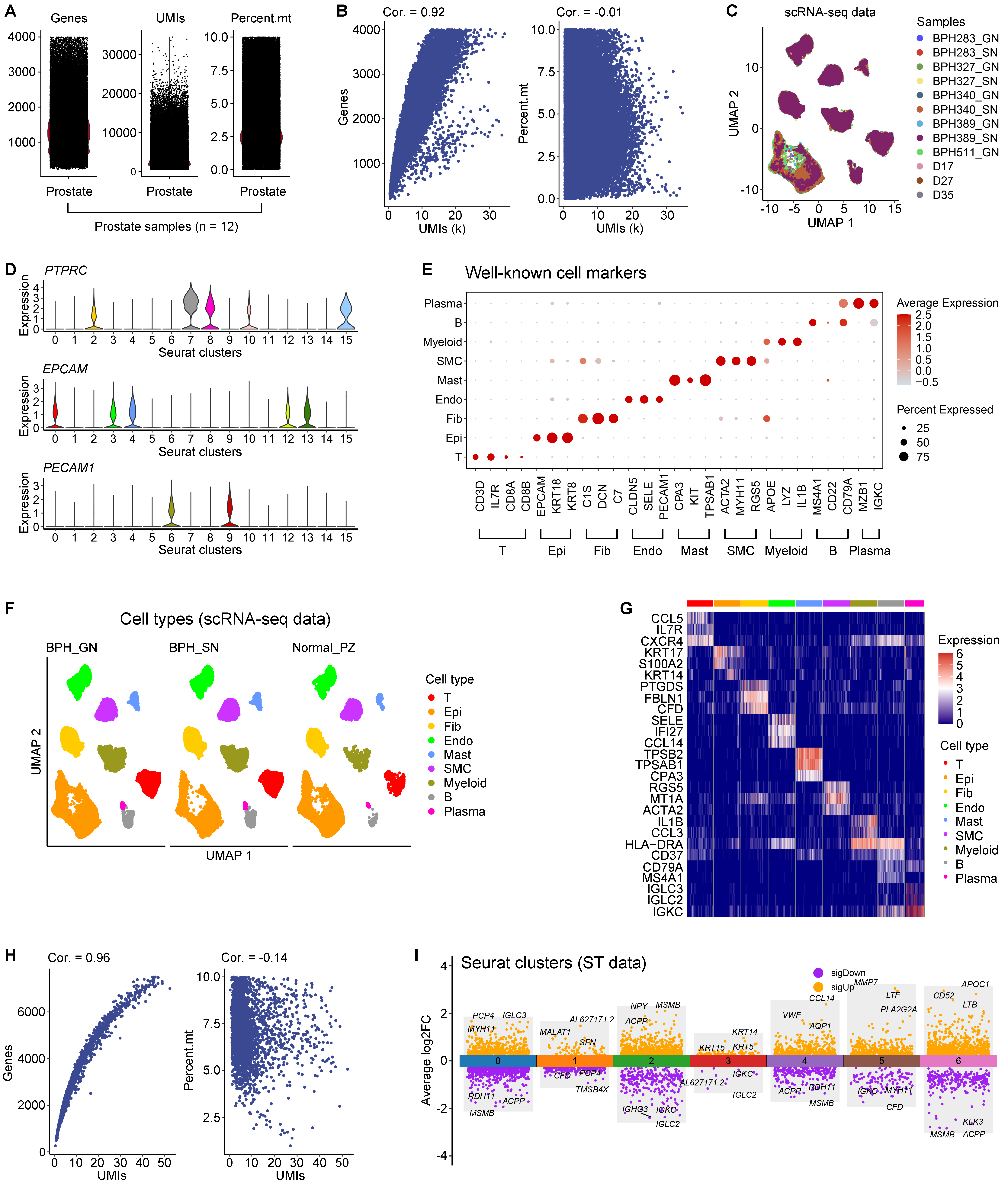


**Supplementary Figure S1.** The atlas of single-cell and spatial transcriptomics in prostate tissues (n=13). (A-G) General information for scRNA-seq data: **(A)** Violin plots depicting the distribution of UMIs, genes, and the proportion of mitochondrial gene expression relative to total gene expression within individual cells (percent.mt); **(B)** Correlations between genes and UMIs as well as the association between percent.mt and UMIs; **(C)** The UMAP visualization of the distribution patterns of the 12 samples; **(D)** Violin plots depicting the expression levels of *PTPRC*, *EPCAM*, and *PECAM1* across distinct Seurat clusters; **(E)** Bubble plots of the marker genes expressed in the major cell types. Dot color reflects expression level and dot size represents the percent of cells expressing marker genes in different cell types; **(F)** UMAP plot depicting the distribution of 84,835 cells across each group. Each cell type is shown in different color; **(G)** Heatmap depicting the expression levels of the top three characteristic genes in each specific cell type. (H-I) General information for ST data: **(H)** Correlations between genes and UMIs as well as the association between percent.mt and UMIs; **(I)** Volcano plot depicting the expression levels of the characteristic genes in each specific spacial cluster.


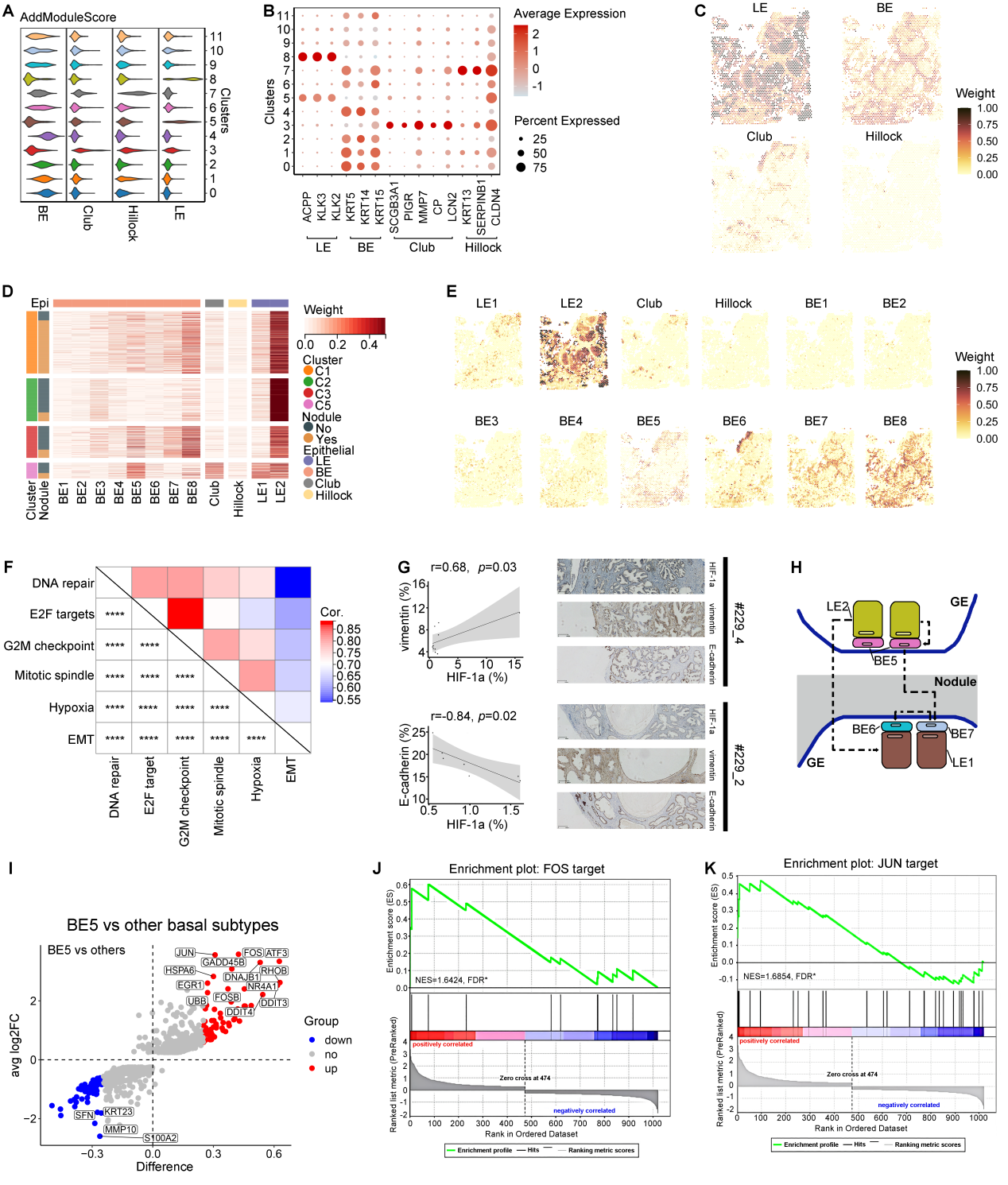


**Supplementary Figure S2**. Subsets analysis of epithelial cells. **(A)** Violin plots depicting the AddModuleScore of LE, BE, Club, and Hillock cell signature gene sets across 12 epithelial cell clusters in scRNA-seq data. **(B)** Bubble plots of the marker genes expressed in the major epithelial cell subgroups in scRNA-seq data. Dot color reflects expression level and dot size represents the percent of cells expressing marker genes in different epithelial cell subtypes. **(C)** Weight of LE, BE, Club, Hillock cells in BPH tissue slide. **(D)** Heatmap displaying the weight of each epithelial cell subgroup for epithelial clusters in ST data. **(E)** Weight of 12 epithelial cell subgroups in BPH tissue slide. **(F)** Heatmaps illustrating pearson correlations among the singscores of Hallmark signaling pathways in epithelial cells using scRNA-seq data. **(G)** Scatter pot depicting the pearson correlation between the percentage of positive (100-negative) Vimentin expression and HIF-1a expression, as well as the percentage of positive (100-negative) E-cadherin expression and HIF-1a expression; Representative images of vimentin, E-cadherin, and HIF-1a expression in BPH tissues; Scale bar: 200μm. **(H)** Schematic diagram illustrating the developmental branch from LE2 subgroup to BE6 subgroup. **(I)** Volcano plot depicting the expression levels of the differentially expressed genes (DEGs) between BE5 versus other BE cells. **(J-K)** Enrichment plots depicting the enrichment scores of JUN and FOS target factors gene sets in BE5 cells compared to other BE cells. NES: Normalized Enrichment Score.


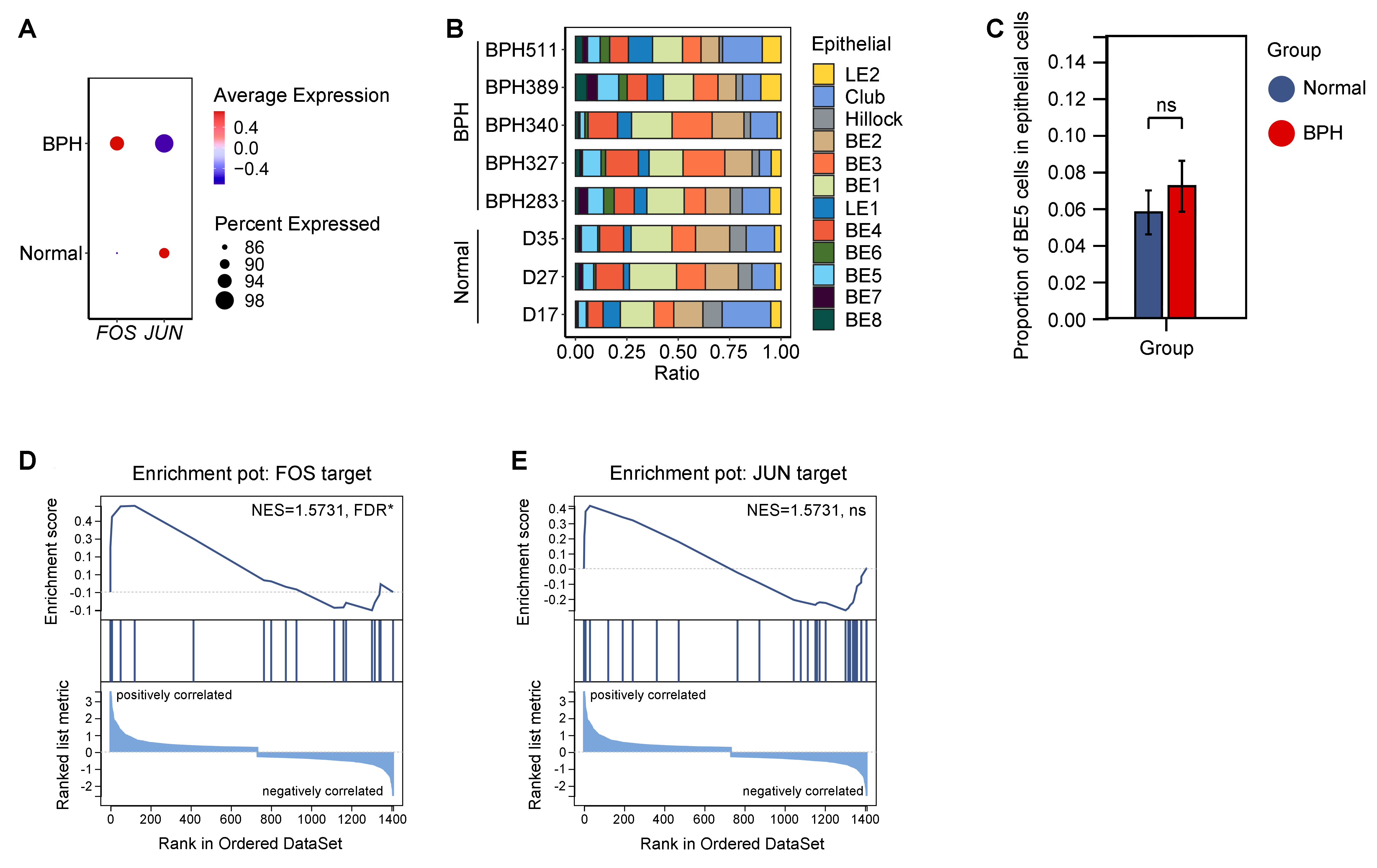


**Supplementary Figure S3**. Characteristic of BE5 subgroup in BPH tissue. **(A)** Bubble plots of *FOS* and *JUN* expressed in BE5 cells of normal and BPH tissues in scRNA-seq data. Dot color reflects expression level and dot size represents the percent of cells expressing marker genes in different groups. **(B)** Ratio of epithelial subgroups in each sample. **(C)** Bar plot illustrating the proportion of BE5 cells in epithelial cells in both BPH and normal prostate tissues. **(D-E)** Enrichment plots depicting the enrichment scores of JUN and FOS target factors gene sets in BE5 cells compared to other BE cells within BPH tissues. NES: Normalized Enrichment Score. ns: FDR>0.05 or *p* value>0.05.


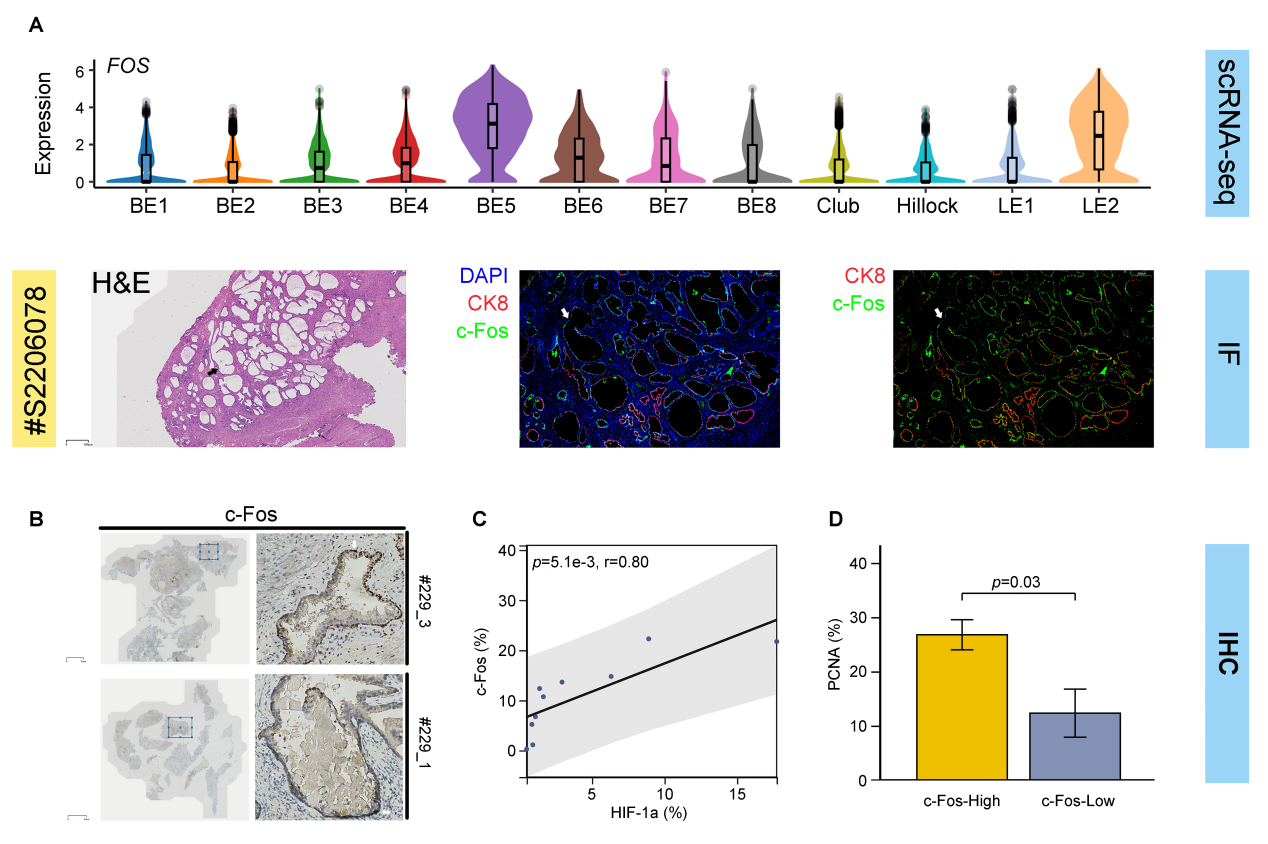


**Supplementary Figure S4**. Localization and expression of *FOS* within BPH tissues. **(A)** Violin plots depicting the expression of *FOS* across 12 epithelial cell subgroups in scRNA-seq data (up); analysis of c-Fos and Cytokeratin 8 (CK8) expression by anti-c-Fos and anti-Cytokeratin 8 immunofluorescence (green and red) in prostate tissues of BPH patients (nuclei counterstained with DAPI) (down). **(B)** IHC staining. Representative images of c-Fos expression in BPH tissues. **(C)** Scatter pot depicting the pearson correlation between the percentage of positive (100-negative) c-Fos expression and HIF-1a expression. **(D)** Bar plot illustrating the percentage of positive (100-negative) PCNA expression in both c-Fos high-expression and c-Fos low-expression BPH patients; The mean value of the percentage of positive (100-negative) c-Fos expression served as the threshold to classify patients with BPHinto two groups: those with high-expression and low-expression levels of c-Fos.


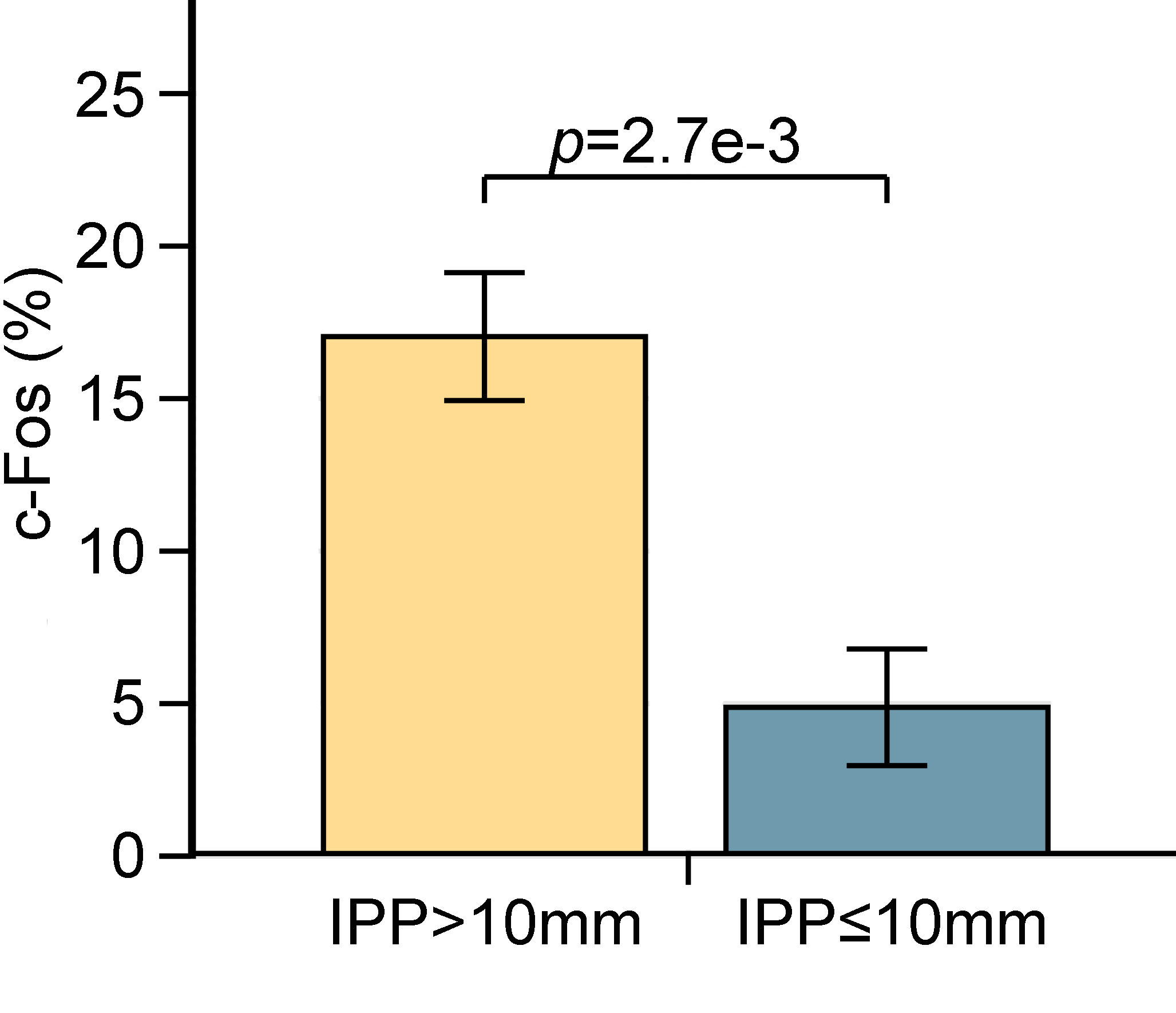


**Supplementary Figure S5**. Bar plot illustrating the percentage of positive (100-negative) c-Fos expression in both IPP>10mm and IPP≤10mm BPH patients.


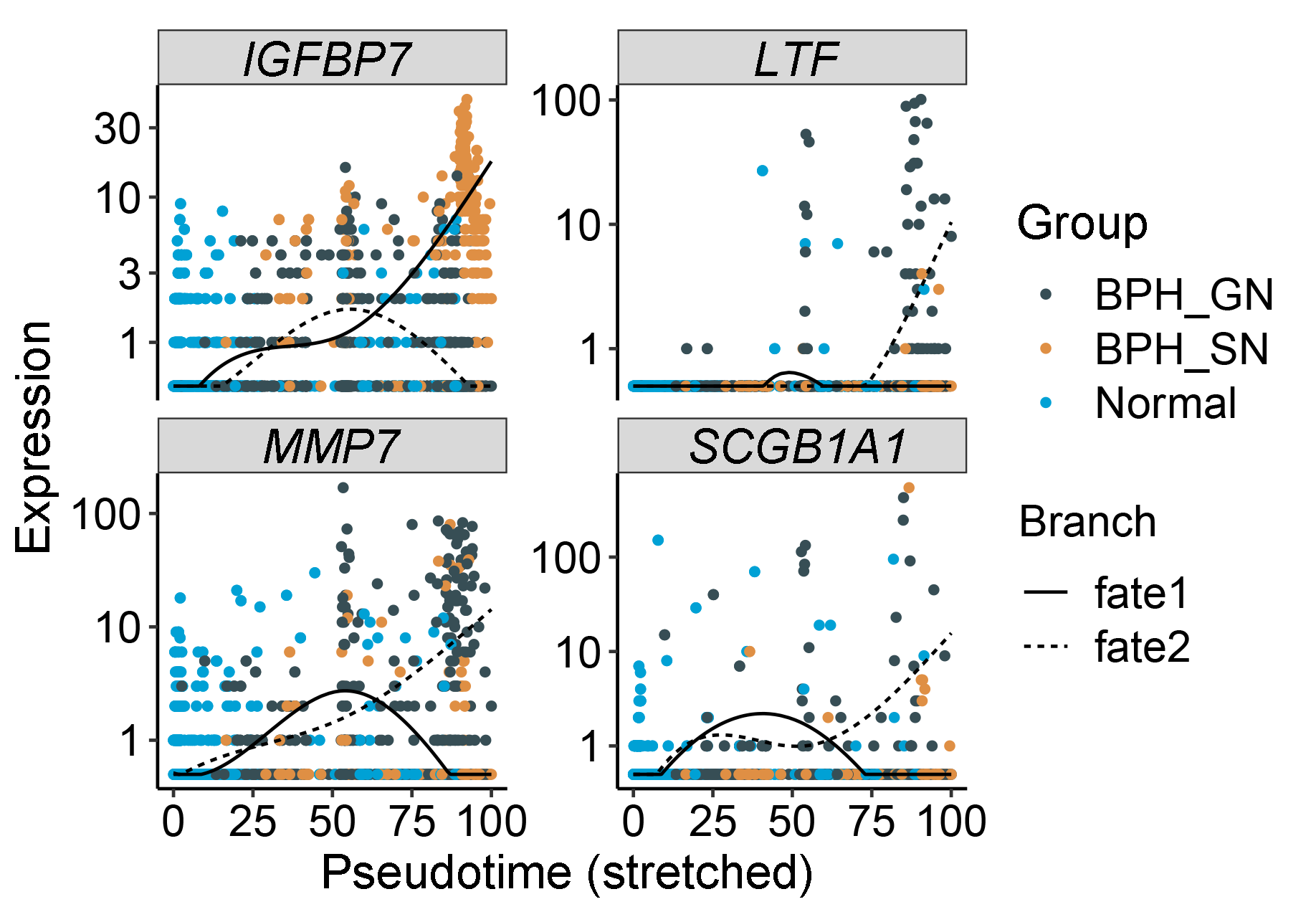


**Supplementary Figure S6**. Dot plots of dynamic expression of top four DEGs along two cell fates at the branch point 3 in the pseudo-time cell trajectory for the normal, BPH_GN, and BPH_SN BE5 cells.
